# Inotropic score and vasoactive inotropic score as predictors of outcomes in congenital diaphragmatic hernia: A single center retrospective study

**DOI:** 10.3389/fped.2023.1101546

**Published:** 2023-02-01

**Authors:** Srirupa Hari Gopal, Cynthia L. Toy, Morcos Hanna, Betul Yilmaz Furtun, Joseph L. Hagan, Ahmed A. Nassr, Caraciolo J. Fernandes, Sundeep Keswani, Sharada H. Gowda

**Affiliations:** ^1^ Section of Neonatology, Department of Pediatrics, Baylor College of Medicine/Texas Children's Hospital, Houston, TX, United States; ^2^ Department of Pharmacy, Texas Children's hospital, Houston, TX, United States; ^3^ Section of Pediatric Cardiology, Department of Pediatrics, Baylor College of Medicine/Texas Children's Hospital, Houston, TX, United States; ^4^ Department of Obstetrics and Gynecology, Maternal Fetal Medicine/Fetal Intervention Baylor College of Medicine, Houston, TX, United States; ^5^ Department of Pediatric Surgery, Baylor College of Medicine/Texas Children's Hospital, Houston, TX, United States

**Keywords:** congenital diaphragmatic hernia, pulmonary hypertension, ventricular dysfunction, inotropic score, vasoactive inotropic score, extracorporeal membrane oxygenation

## Abstract

**Background:**

Neonates with congenital diaphragmatic hernia (CDH) have varying degrees of pulmonary hypoplasia, pulmonary hypertension (PH) and cardiac dysfunction. These neonates frequently require vasoactive support and are at high risk for mortality and morbidity, including prolonged ventilator support, need for extracorporeal membrane oxygenation (ECMO), prolonged length of stay, and need for tracheostomy. However, identifying which infants are at increased risk can be challenging. In this study, we sought to investigate the utility of the inotropic score (IS) and vasoactive inotropic score (VIS) as tools to predict significant clinical outcomes and overall survival in patients with CDH. Additionally, we evaluated the correlation between IS/VIS and postnatal echocardiographic variables.

**Methods:**

This was a retrospective chart review of 57 patients with CDH whose postnatal care was based on a standardized institutional protocol. We calculated the IS/VIS at 6-, 12-, 24-, 48 hours of life (HOL), on the day of CDH repair and 24- and 48 hours after surgical repair. The association of these scores with postnatal echocardiographic markers was analyzed using Pearson's correlation and linear regression, while logistic regression was used for binary outcomes, and Cox proportional hazards regression was used to assess associations with survival.

**Results:**

We found that every one-unit increase in IS/VIS at 6 HOL was associated with 13% increase in the odds of ECMO (*p* = 0.034) and 10.1% increase in risk of death (*p* = 0.021). An increase in IS/VIS at 12-, 24- and 48-HOL was associated with posterior septal bowing in the first postnatal echocardiogram (*p* < 0.05 for all). Additionally, we noted an inverse relationship between IS (*r* = −0.281, *p* = 0.036) and VIS (*r* = −0.288, *p* = 0.031) on the day of repair and left ventricle (LV) systolic function in first postnatal echocardiogram. Increase in IS (*r* = −0.307, *p* = 0.024) and VIS (*r* = −0.285, *p* = 0.037) on the day of repair was associated with decreased LV function on the post-repair echocardiogram.

**Conclusion:**

This retrospective study showed a significant association between IS/VIS obtained at various time points with clinical outcomes and echocardiographic findings in CDH, which could be used to guide prognosis and management in this patient population.

## Introduction

Congenital diaphragmatic hernia (CDH) is a congenital defect that presents with varying degrees of pulmonary hypoplasia, pulmonary hypertension and ventricular dysfunction, which play significant roles in the overall morbidity and mortality (1, 2). Despite advances in clinical and surgical management of CDH, the overall morbidity and mortality remain high (3). The trifecta of pulmonary hypertension, pulmonary hypoplasia and myocardial dysfunction in CDH leads to hypoxic respiratory failure and circulatory insufficiency, which, when left untreated, can progress to end organ hypoperfusion and shock. This multiorgan dysfunction contributes to need for prolonged ventilation and need for circulatory support in the form of inotropes and ECMO (4, 5).

In critically ill patients, utilizing scoring indices that accurately reflect severity of illness have been shown to be valuable by providing guidance to bedside clinical management and patient care research (6, 7). Congenital heart surgeons and cardiac critical care physicians use the inotrope score (IS), vasoactive-inotrope score (VIS) and total inotrope exposure score (TIES) to correlate postoperative hemodynamics following surgical repairs (8–11). The VIS obtained at 24 h after admission to a cardiac ICU has been shown to be strongly associated with morbidity and mortality in infants undergoing cardiac surgery (11).

With the interplay of complex physiology that exists in the CDH population, we hypothesized that the inotropic score and vasoactive inotropic score would be useful and effective bedside tools to predict clinical outcomes in CDH. To the best of our knowledge, no such studies have been conducted in the CDH population.

Our primary objective with this study was to determine the correlation of the inotropic and vasoactive inotropic scores at 6-, 12-, 24-, 48-HOL, on the day of CDH repair and 24- and 48 hours following repair with various clinical outcomes and postnatal echocardiographic parameters. Inotropic score (IS) and vasoactive inotropic scores (VIS) are calculated as shown below (8):

Inotropicscore(IS)=Dopamine(mcg/kg/min)+Dobutamine(mcg/kg/min)+100×Epinephrine(mcg/kg/min)

VasoactiveInotropicScore(VIS)=IS+10×Milrinone(mcg/kg/min)+10,000×Vasopressin⁡(units/kg/min)+100×Norepinephrine(mcg/kg/min)

## Materials and methods

### Setting and infrastructure

This single center study was conducted in a 187 bed NICU of a quaternary care children's hospital, which is a CDH/ECMO referral center. All patients were treated on a standardized protocol. Following approval from the Baylor College of Medicine Institutional Review Board (IRB # H-49059), data was extracted from the medical records, and stored in encrypted folders on secure institutional servers.

### Study design

This retrospective cohort study included neonates ≤28 days of age admitted to the level IV NICU between January 2017 and December 2021 with a diagnosis of CDH, and who received one or more inotropic medications (including dopamine, dobutamine, epinephrine, vasopressin, milrinone, norepinephrine and hydrocortisone) during their hospital stay. Our exclusion criteria consisted of patients with critical congenital heart disease (defined as ductal dependent systemic and pulmonary circulation requiring prostaglandin E1 (PGE) or cardiac intervention within first few days of life), multiple congenital anomalies, and lethal genetic abnormalities who underwent redirection of care within 48 h of life.

### Data collection

Baseline demographics, clinical, and outcomes data were obtained. Demographic data included gestational age, birthweight, sex, and APGAR scores at 1- and 5 min. Clinical data included fetal CDH measurements {Observed/Expected Total Fetal Lung Volume (O/E TFLV), Lung-Head Ratio (LHR) and percent liver herniation (%LH)}, severity of hernia (based on prenatal CDH measurements described by Ruano et al.) (12), side of hernia, fetal intervention such as Fetal Tracheal Balloon Occlusion (FETO), and inotropic medications used. Postnatal use of vasoactive medications was obtained for different time points: 6-, 12-, 24-, 48-HOL, on the day of CDH repair and 24- and 48 h after surgical repair. This data was extracted and screened by 2 study personnel (SHaG and CT) to ensure accuracy. We obtained echocardiographic data from the first postnatal study, following CDH surgical repair, and at 28 days of life. Echocardiographic measures included data on septal defects (presence of atrial septal defect(s) (ASD), ventricular septal defect(s) (VSD) and/or patent foramen ovale (PFO)), ASD/VSD shunt direction, tricuspid regurgitation (TR) velocity, ventricular septal position, patent ductus arteriosus (PDA) size (small, moderate and large), PDA shunt direction (left to right, right to left or bidirectional), qualitative right and left ventricular function (mild, moderate or severely depressed) and ejection fraction of LV based on American Society for Echocardiography (ASE) guidelines (13). Primary clinical outcomes were use of ECMO and mortality, and secondary clinical outcomes were duration of ECMO, length of hospital stay and postnatal echocardiographic markers.

### IS/VIS calculations

The inotropic score and vasoactive inotropic scores were calculated (using the formulas above) for each of the following time points: 6-, 12-, 24-, 48-HOL, on the day of CDH repair and 24- and 48 hours.

### Clinical endpoints and outcomes

Following data extraction, we analyzed IS/VIS at different time points with echocardiographic data and clinical outcomes. We performed the following analyses:

1. Associated IS/VIS at 6-, 12-, 24- and 48-HOL, on the day of repair and 24- and 48 hours following repair with primary and secondary outcomes measures.
2. Associated 6-, 12-, 24- and 48-HOL IS/VIS with echocardiographic measures obtained in first postnatal echocardiogram.
3. Associated IS/VIS on the day of repair and 24- and 48 hours following repair with first postnatal, post repair and 28-day echocardiographic measurements.

### Statistical analysis

Logistic regression and receiver operating characteristic (ROC) curve analyses were used to investigate associations of IS/VIS with binary outcomes, while Pearson's correlation and linear regression analysis were used for continuous outcomes and Cox proportional hazards regression was used to assess associations with survival time. Multiple logistic regression analysis was used to examine associations of IS/VIS with ECMO, after controlling for CDH severity. SAS version 9.4 (SAS Institute Inc., Cary, North Carolina) was used for data analysis. A two-sided 5% significance level was used for hypothesis tests.

## Results

### Cohort characteristics

**Figure 1:**
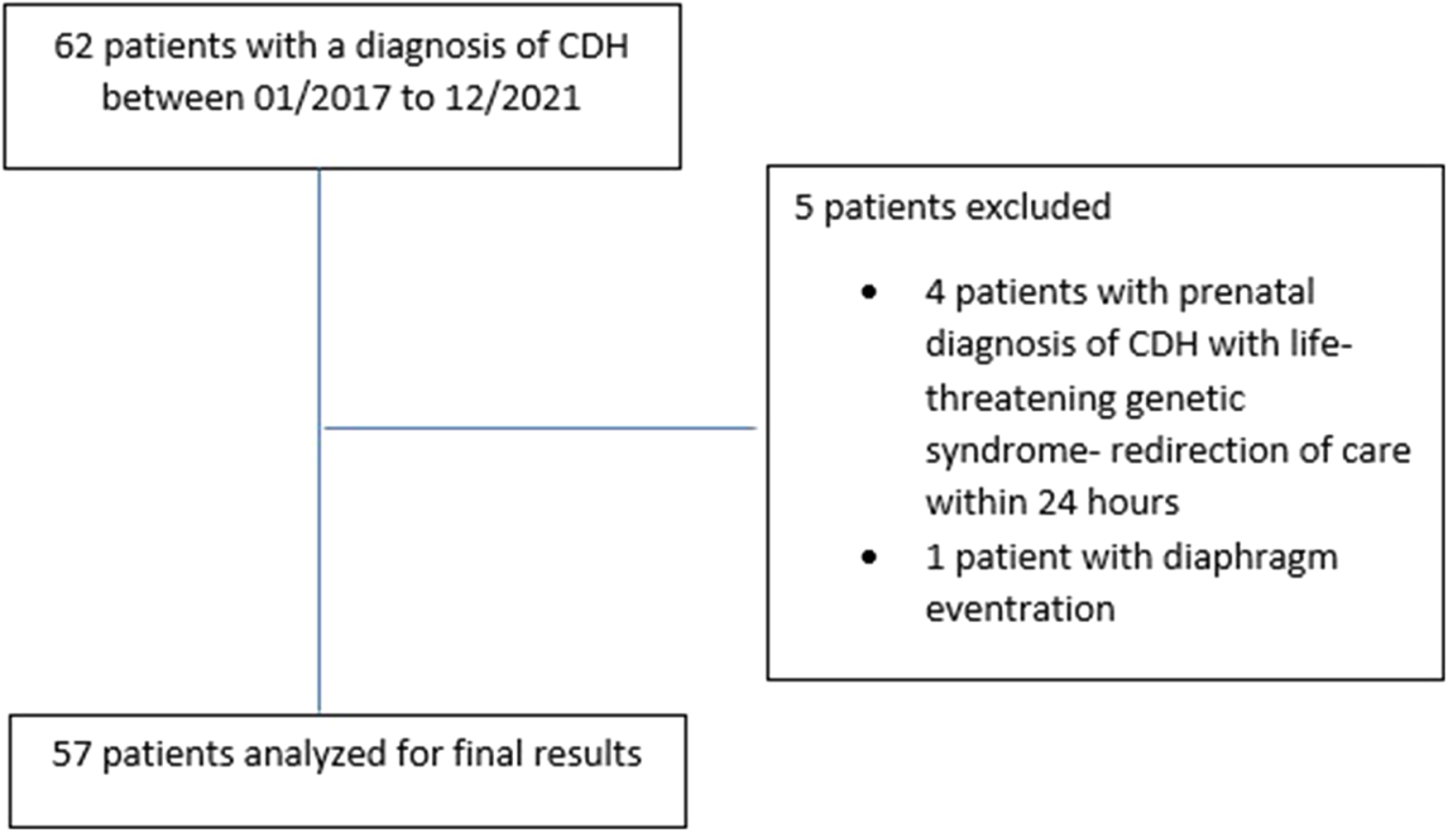
Study characteristics-inclusion and exclusion of patients.

After excluding 4 patients with lethal genetic syndrome who had redirection of care within the 24 h of birth and 1 patient with diaphragmatic eventration, 57 patients were included in the final study cohort (Figure 1).

Patient characteristics are shown in Table 1. Based on prenatal imaging, 28%, 40%, and 30% of the cohort had mild, moderate and severe disease, respectively (12). Prenatal imaging was not available for one out-born patient who had a postnatal diagnosis of CDH. Twenty-six percent of patients in the cohort had prenatal CDH intervention in the form of Fetal Endoscopic Tracheal Occlusion (FETO). The primary inotropic therapy was dopamine in 91% of the patients. Fifty-three percent of the patients were treated with hydrocortisone by 24 h of life for the management of hypotension, after escalation of dopamine therapy per institutional guidelines. Forty percent required ECMO. Overall, 77% of all patients survived to discharge. Out of the twelve patients who died, ten required ECMO. Four died on ECMO-Two of which were due to bleeding on ECMO. Four Babies died following decannulation due to cardiorespiratory decompensation. Two babies died a few weeks after being on ECMO due to sepsis.

**Table 1:** Patient characteristics.

Characteristic	*n* = 57
**Sex** ^1^	
Female	23 (40.4)
Male	34 (59.7)
**Maternal race** ^1^	
Asian	5 (8.8)
Black	8 (14.0)
White	44 (77.2)
Hispanic ethnicity^1^	17 (29.8)
Gestational age (weeks)^2^	38.1 (36.3, 39.1)
Birth weight (g)^2^	2,940 (2625, 3190)
Apgar score at 1 minute^2^	4 (2, 6)
Apgar score at 5 minutes^2^	7 (5, 8)
O/E TFLV^2^	0.270 (0.225, 0.430)
LH%^2^	0.190 (0.050, 0.285)
**CDH severity** ^1^	
Mild	16 (28.1)
Moderate	23 (40.4)
Severe	17 (29.8)
Missing	1 (1.8)
FETO^1^	15 (26.3)
**Hernia side** ^1^	
Left	48 (84.2)
Right	6 (10.5)
Bilateral	3 (5.3)
**Final disposition** ^1^	
Died	12 (21.1)
Home	44 (77.2)
Transferred	1 (1.8)
Hydrocortisone by 24 h of life^1^	30 (52.6)
Dopamine as initial vasoactive agent^1^	52 (91.2)
Need for ECMO^1^	23 (40.4)

^1^ Frequency (%).

^2^ Median (interquartile range).

O/E TFLV, observed/expected total fetal lung volume; LH%, percentage liver herniation; FETO, fetal endoscopic tracheal occlusion; ECMO, extracorporeal membrane oxygenation.

### VIS/is for predicting primary outcomes

IS and VIS at 6 HOL were significantly (*p* = 0.034) associated with ECMO (Table 2). Every one-unit increase in IS and VIS at 6-HOL was associated with a 13% increase in the odds of ECMO (OR: 1.130, 95% CI: 1.010–1.264). Using an IS and VIS at 6-HOL cut-off of ≥7.5 to predict ECMO yielded the highest sensitivity and specificity average (sensitivity = 39.1%, specificity = 88.2%). After controlling for CDH severity, IS and VIS at 6-HOL were still significantly (*p* = 0.045) associated with ECMO (adjusted OR: 1.138, 95% CI: 1.003, 1.291, 66.9% area under ROC curve). Additionally, we found that every one-unit increase in IS and VIS at 6 HOL was associated with a 10.1% increase in risk of death (hazard ratio = 1.101, 95% CI: 1.014–1.194, *p* = 0.021) (Table 2).

**Table 2:** IS/VIS obtained in the first 48 HOL as a predictor for need for ECMO and death.

Outcome	Predictor	Area under ROC curve	*p*-value
Need for ECMO	IS at 6 h	60.1%	0.034*
	VIS at 6 h	60.1%	0.034*
	IS at 12 h	56.0%	0.263
	VIS at 12 h	56.0%	0.272
	IS at 24 h	50.2%	0.649
	VIS at 24 h	50.6%	0.608
	IS at 48 h	57.3%	0.353
	VIS at 48 h	55.8%	0.454
Death	Predictor	Hazard Ratio (95% CI)	*p*-value
	IS at 6 h	1.101 (1.014, 1.194)	0.021*
	VIS at 6 h	1.101 (1.014, 1.194)	0.021*
	IS at 12 h	0.983 (0.887, 1.090)	0.749
	VIS at 12 h	0.983 (0.886, 1.090)	0.741
	IS at 24 h	0.904 (0.789, 1.035)	0.142
	VIS at 24 h	0.913 (0.802, 1.040)	0.173
	IS at 48 h	0.894 (0.757, 1.055)	0.185
	VIS at 48 h	0.902 (0.767, 1.061)	0.212

* Indicates statistically significant association.

### IS/VIS and its correlation with echocardiographic measures

We correlated the IS/VIS obtained at various time points in the first 48 h of life (6-, 12-, 24- and 48-HOL) with echocardiographic markers of pulmonary hypertension and ventricular function seen in the first postnatal echocardiogram. We found that early increased VIS/IS was associated with worsening pulmonary hypertension.

We found that IS/VIS obtained at 6-HOL was associated with increased odds of bidirectional atrial level shunting, with every one-unit increase in IS/VIS at 6-HOL associated with 13.9% increase in the odds of bidirectional atrial level shunting (odds ratio (OR) = 1.139, 95% CI: 1.007–1.289, *p* = 0.038). We also noted that higher VIS at 12-HOL was significantly associated with bowing of interventricular septum (IVS) (OR: 1.135, 95% CI: 1.019–1.265, *p* = 0.021), higher IS at 12-HOL was significantly associated with IVS bowing (OR: 1.136, 95% CI: 1.019–1.265, *p* = 0.021) and higher IS and VIS at 24 hours were associated with bowing IVS (OR: 1.167, 95% CI: 1.038–1.313, *p* = 0.010 for both). Higher VIS at 48 hours were significantly associated with bowing IVS (OR: 1.140, 95% CI: 1.002, 1.298, *p* = 0.047). There was an inverse relationship between in IS/VIS at 6 HOL (OR: 0.896, 95% CI: 0.804–0.999, *p* = 0.048) 12 HOL (IS OR: 0.894, 95% CI: 0.810–0.986, *p* = 0.026, VIS OR: 0.895, 95% CI: 0.811–0.987, *p* = 0.027) and 24-HOL (IS OR: 0.897, 95% CI: 0.811–0.991, *p* = 0.033, VIS OR: 0.898, 95% CI: 0.813–0.993, *p* = 0.036) with septal flattening, with higher scores associated with lower odds of septal flattening. The associations of IS/VIS obtained in first 48-HOL with first postnatal echocardiogram is shown in Table 3.

**Table 3:** Associations of iS/VIS obtained in first 48-HOL with echocardiographic markers in 1st postnatal echocardiogram.

Echocardiographic marker	Predictor	Area under ROC curve	*p*-value
Atrial level shunting	IS at 6 h	62.5%	0.038*
	VIS at 6 h	62.5%	0.038*
	IS at 12 h	57.4%	0.209
	VIS at 12 h	57.4%	0.215
	IS at 24 h	58.2%	0.232
	VIS at 24 h	58.1%	0.238
	IS at 48 h	58.4%	0.305
	VIS at 48 h	59.7%	0.240
Intraventricular Septal bowing	IS at 6 h	57.2%	0.194
	VIS at 6 h	57.2%	0.194
	IS at 12 h	75.3%	0.021*
	VIS at 12 h	75.3%	0.021*
	IS at 24 h	74.4%	0.010*
	VIS at 24 h	74.5%	0.010*
	IS at 48 h	69.0%	0.072
	VIS at 48 h	72.1%	0.047*
Intraventricular Septal flattening	IS at 6 h	63.0%	0.048*
	VIS at 6 h	63.0%	0.048*
	IS at 12 h	69.4%	0.026*
	VIS at 12 h	69.4%	0.027*
	IS at 24 h	68.4%	0.033*
	VIS at 24 h	67.9%	0.036*

* Indicates statistically significant association.

**Figure 2:**
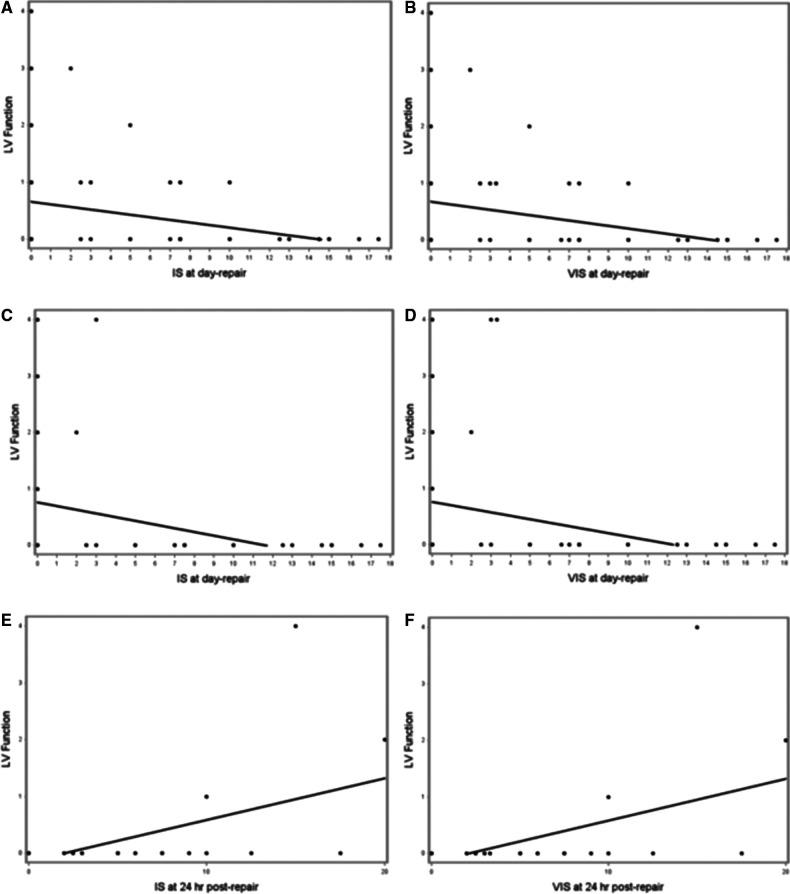
(**A–F**). Scatterplots of IS and VIS on the day of repair vs. LV function in first postnatal echocardiogram (**A** and **B**, respectively), IS and VIS on the day of repair vs. LV function in post-repair echocardiogram (**C** and **D**, respectively), and IS and VIS at 24 h following repair vs. LV function in 28-day echocardiogram (**E** and **F**, respectively).

When examining associations of IS/VIS obtained at various time points following the CDH repair (day of repair, 24- and 48 hours after repair) with the first post-natal, post-repair and 28-day echocardiogram (Table 4), we noted that for every one-unit increase in the IS at day of repair the predicted LV function at first post-natal Echocardiogram decreased by 0.045 (*r* = −0.281, *p* = 0.036, Figure 2A). Similarly, for every one-unit increase in the VIS at day of repair the predicted LV function at first post-natal ECHO decreased by 0.047 (*r* = −0.288, *p* = 0.031, Table 4, Figure 2B). Additionally, we found that VIS on day of repair explained 8.1% of the variation in LV function, with the predicted LV function on the post-repair echocardiogram decreasing by 0.062 for every one-unit increase in VIS on day of repair (*r* = −0.285, *p* = 0.037). Similarly, IS at day of repair explained 9.4% of the variation in LV function with every one-unit increase in IS predicting a 0.065-unit decrease in LV function in the post-repair echocardiogram (*r* = −0.307, *p* = 0.024) (Table 4 and Figures 2C,D). Finally, we found direct relationships between IS/VIS on the day of repair, 24 h following repair and 48 h following repair with LV function in the 28-day echocardiogram. IS and VIS at 24 h post repair explained the largest proportion of the variation in LV function (29.4% and 29.5%, respectively). For every one-unit increase in IS and VIS at 24 h post repair, the predicted LV function increased by 0.073 (*p* < 0.001) and 0.074 (*p* < 0.001), respectively (Table 4 and Figures 2E,F). We found no statistically significant correlation between IS/VIS at different time points with markers of RV function, PDA flow direction and TR velocity.

**Table 4:** Associations of iS/VIS obtained following CDH repair (on the day of repair, 24- and 48 hours after repair) with left ventricular function in 1st postnatal, post-repair and 28-day echocardiogram.

Outcome	Predictor	*r* ^2^	*p*-value
LV function in first post-natal echocardiogram	IS at day-repair	0.079	0.036*
	VIS at day-repair	0.083	0.031*
	IS 24-hour post repair	0.047	0.110
	VIS 24-hour post repair	0.047	0.109
LV function in post-repair echocardiogram	IS at day-repair	0.094	0.024*
	VIS at day-repair	0.081	0.037*
	IS 24 h post repair	0.031	0.204
	VIS 24 h post repair	0.022	0.287
LV function in 28-day echocardiogram	IS at day-repair	0.112	0.035*
	VIS at day-repair	0.110	0.037*
	IS 24 h post repair	0.294	<0.001*
	VIS 24 h post repair	0.295	<0.001*
	IS 48 h post repair	0.117	0.031*
	VIS 48 h post repair	0.115	0.032*

* Indicates statistically significant association.

## Discussion

We found IS and VIS to be a useful tool for assessing patients with CDH. We observed increased IS/VIS obtained at 6- HOL was associated with ECMO and mortality. IS/VIS obtained at different time points correlated well with various echocardiographic markers of PH and ventricular function, thus highlighting their potential utility as bedside tools for clinical assessment and guiding management.

A thorough understanding of the pathophysiology of systemic hypotension in CDH is still evolving. Elevated pulmonary vascular resistance (PVR) and right ventricular (RV) afterload decreases pulmonary blood flow leading to decreased pulmonary venous return and net cardiac output. Recently, ventricular dysfunction has emerged as an important area of clinical significance (14). LV dysfunction seen in this patient population contributes to low cardiac output, which has been reported as an independent predictor for death and ECMO use (14). Our study validated this by demonstrating an increase in IS/VIS at 6-HOL, which was associated with mortality and increased need for ECMO.

**Figure 3:**
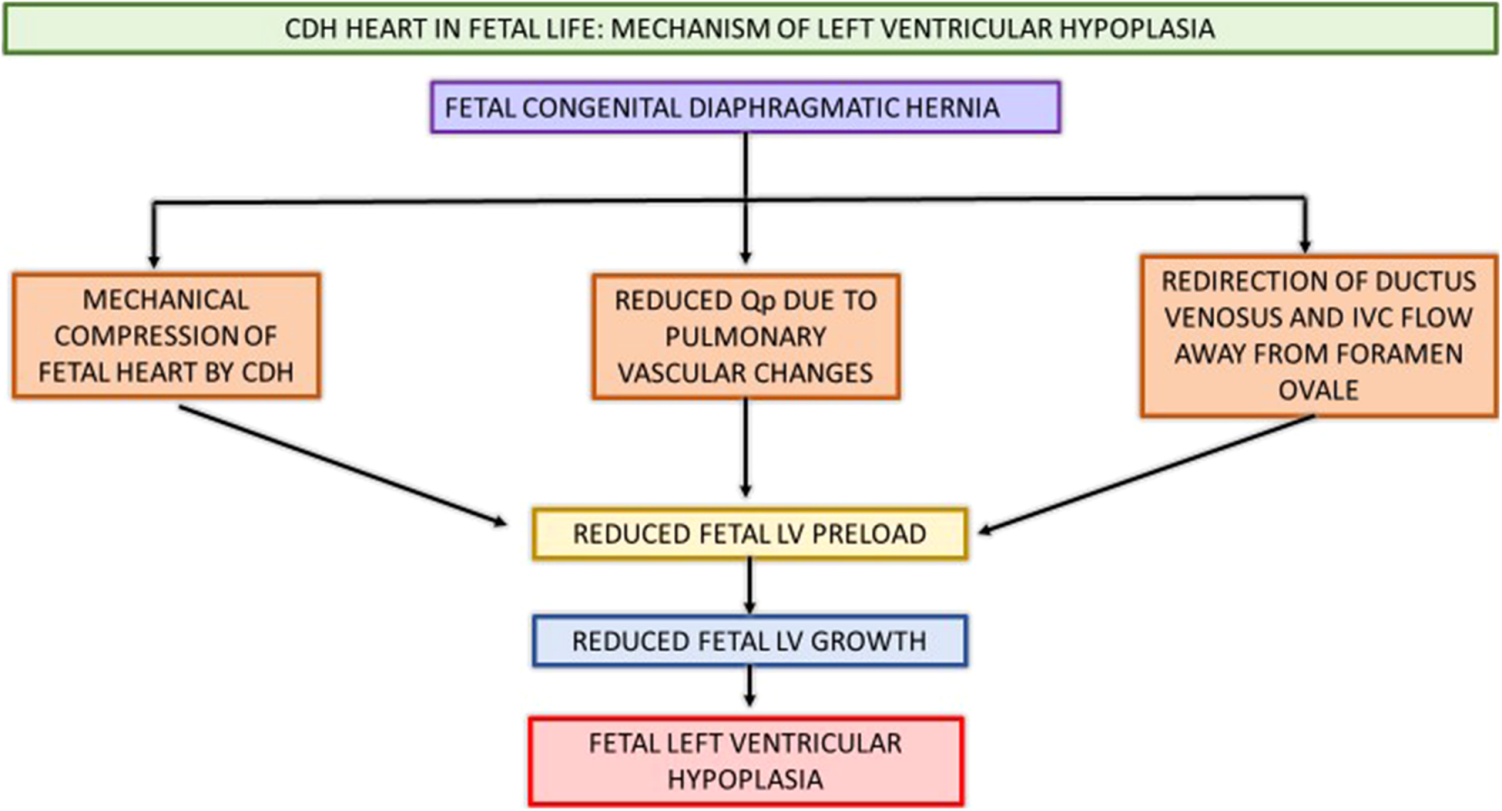
Schematic showing CDH heart in fetal life and pathophysiology contributing to postnatal LV hypoplasia and systemic hypotension. CDH, congenital diaphragmatic hernia; Qp, pulmonary blood flow; IVC, inferior vena cava; LV, left ventricle.

**Figure 4:**
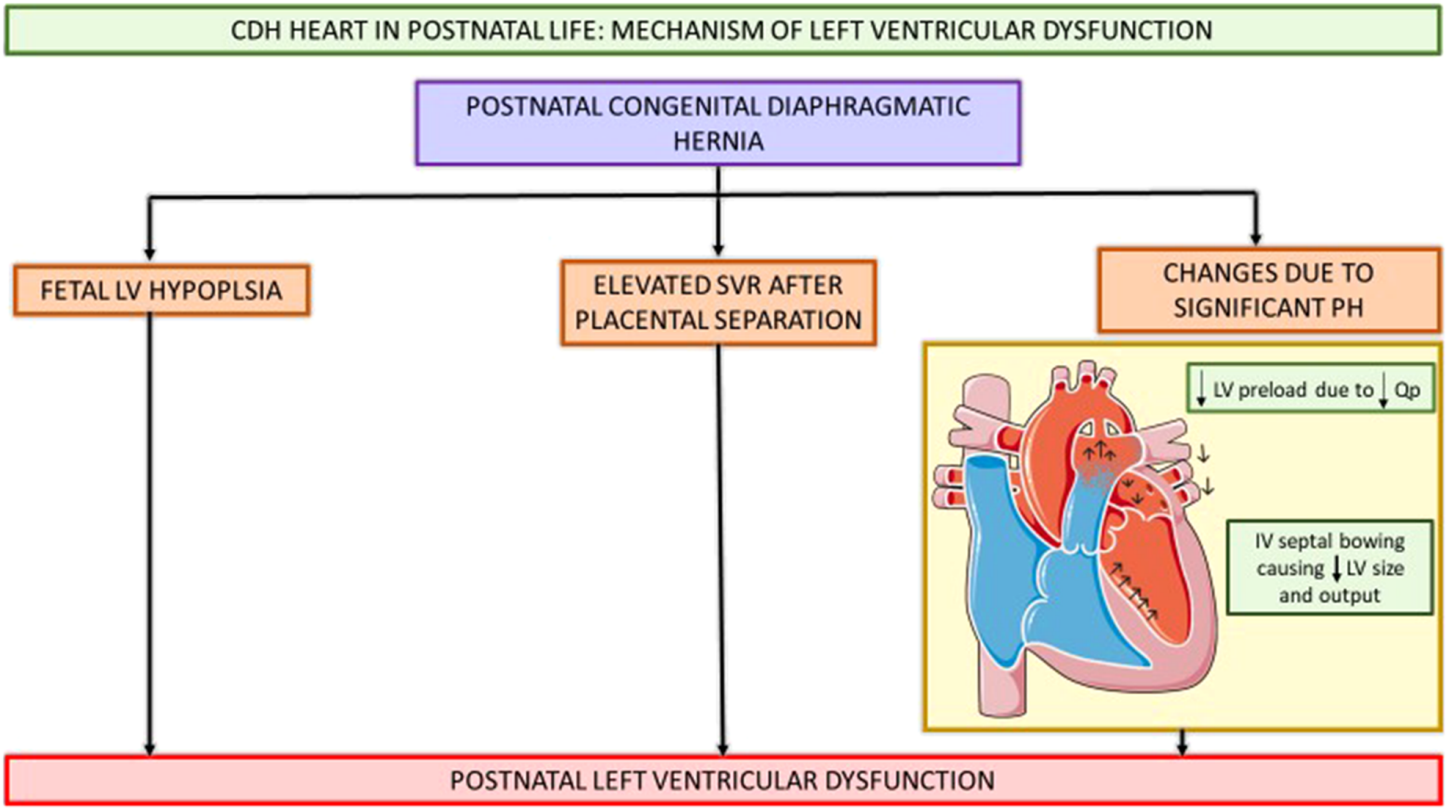
Schematic showing CDH heart in postnatal life and pathophysiology contributing to postnatal LV hypoplasia and systemic hypotension. CDH, congenital diaphragmatic hernia; Qp, pulmonary blood flow; LV, left ventricle; SVR, systemic vascular resistance; IV, intraventricular septum. (This Figure was partly generated using Servier Medical Art, provided by Servier, licensed under a Creative Commons Attribution 3.0 unported license.).

Etiology of LV dysfunction in CDH is incompletely understood. The fetal and post-natal cardiac physiology contributing to LV dysfunction has been summarized in Figures 3, 4.

Various fetal and postnatal mechanisms for LV dysfunction in the CDH population have been hypothesized, such as underdeveloped left sided cardiac structures, increased SVR and ventricular interdependence (15–17). Early fetal echocardiograms show evidence of smaller left heart structures (16, 18). Left ventricular hypoplasia is thought to be a product of mechanical compression of the fetal heart by the herniated contents in the thoracic cavity (19). Re-direction of ductus venosus and inferior vena cava (IVC) flow away from the foramen ovale leads to decreased LV preload and inadequate fetal myocardial growth (15, 20). Additionally, postnatal stress with elevated systemic vascular resistance (SVR) after placental separation further worsens ventricular dysfunction and is associated with adverse postnatal outcomes (14, 16, 21, 22). Thirdly, fetal lung growth is impeded by intrathoracic herniation of abdominal contents causing reduction in total fetal lung volume and pulmonary hypoplasia. This pulmonary vasculature is maladaptive and maldeveloped as a result of this prenatal insult, thus leading to pulmonary hypertension. The resultant reduced pulmonary blood flow in the setting of this pulmonary hypertension causes decreased pulmonary venous return to the left atrium, and ultimately can lead to postnatal systemic hypotension (15, 17). The RV dilation and hypertrophy alter the myocardial contractility as ventricular inter-dependence is compromised due to septal displacement. With inadequate RV contractility and increased RV afterload, a vicious cycle of decreased pulmonary blood flow, with further reduction in pulmonary venous return, and thus lower LV preload with resultant systemic and coronary hypoperfusion and worsening myocardial contractility ensues (15, 17, 23).

In our study, we observed a significant correlation between IS/VIS at 12-, 24- and 48-HOL with septal bowing seen in the first postnatal echocardiogram-with higher scores associated with septal bowing. We also found an inverse relationship between IS/VIS at 6-,12- and 24-HOL with septal flattening, which can be explained by preserved interventricular interaction with septal flattening, as opposed to septal bowing where the LV preload is decreased, and myocardial contractility is altered with geometric distortion of the left ventricle. This results ultimately in decreased stroke volume and cardiac output thus needing additional inotropic support and higher IS/VIS scores.

Our results showed that an increase in IS/VIS on the day of repair was associated with decreased LV function in the first postnatal and post-repair echocardiogram which may be secondary to persistence of fetal cardiac maladaptation and delays in postnatal transition, resulting in increased need for vasoactive support during the repair and increased metabolic demands during the procedure. In addition, our results showed that an increase in IS/VIS correlated with increase in LV function in the 28-day echocardiogram. This can be explained by the fact that after CDH repair, the mediastinum and cardiac structures shift into anatomically appropriate positions, improving venous return and optimizing preload, permitting recovery of LV function and remodeling of myocardium, all of which contribute to the improved function noted on the echocardiogram obtained after 28 days of life.

The IS/VIS have been validated as a clinical measure of cardioactive medication use in both the adult and pediatric population (8, 10, 24, 25). They have been specifically studied in the cardiac ICU as a predictor for adverse outcomes such as prolonged length of stay, duration of mechanical ventilation, mechanical circulatory support, renal replacement therapy and death (8). Studies in the neonatal population have been emerging and have shown promising results on the clinical use of these scoring tools (26, 27). These studies specifically looked at the preterm population and calculated the VISmax (calculated as the maximum score during birth hospitalization). Aziz et al. reported VISmax to have significant utility to predict mortality in the preterm population, with VISmax >30 associated with universal mortality (26). Kharrat et al. reported the VISmax during the first 12-, 24- and 48 hours of treatment in preterm infants and concluded that a VISmax score ≥20 within 48 hours of treatment initiation was associated with adverse outcomes (27).

### Strengths

We report that IS and VIS are useful prognostic variables in the CDH patient population, where there is an increased need for inotropic support and associated adverse outcomes, such as mortality and ECMO. We add a unique perspective by correlating these scoring modalities with specific echocardiographic markers in neonates with CDH. Additionally, we have taken both pre- and post-repair CDH physiologies into consideration by obtaining serial IS/VIS measurements up to 48 hours of life and 48 h following surgical repair. This provides a temporal understanding of changing CDH physiology from birth until after surgical repair, thus validating the contribution of ventricular remodeling and improvement in cardio-pulmonary physiology following repair in CDH.

### Limitations

One of the major limitations of the study is the retrospective nature of the study due to which the specific timing of echocardiogram, timing, rate of escalation, dosing and type of vasopressor support was not standardized. Additionally, since the study focused specifically on inotropic use, we could not account for other management strategies for systemic hypotension such as use of fluid boluses, blood transfusions and/or use of hydrocortisone, which may have impacted the overall clinical picture of this patient population. We could also not account for contributors of systemic hypotension such as pulmonary vasodilators and/or sedation medications. VIS and IS ≥ 7.5 at 6-HOL had high specificity but low sensitivity in predicting ECMO. Thus, scores <7.5 at 6-HOL could be useful for identifying patients at low risk for ECMO, but other prognostic variables should be investigating to discriminate those who will versus will not receive ECMO among those with VIS and IS ≥ 7.5 at 6-HOL. Predictive accuracy for ECMO decreases after 6-HOL but it is fortunate that the highest predictive accuracy occurs early when it is most useful. Examining predictive accuracy at numerous time points increases the probability of Type I Error (i.e., concluding there is an association with IS/VIS where there really is not). Therefore, findings from this study should be considered exploratory and future prospective studies should focus on IS/VIS at 6-HOL and within the first day of repair. Despite these limitations, this is one of the first studies validating the use of these scoring tools in predicting outcomes and correlating with echocardiographic markers in the CDH population.

### Future research

Our goal with this study is to stimulate further research into the use of IS/VIS following its validation in the CDH population. This data has now laid the foundation for a potential pilot prospective model to further investigate these scoring tools in a standardized protocol-based setting and help predict various short-term and long-term clinical outcomes. This would further add to the evidence and encourage the use of these scoring modalities in the CDH population. Additionally, it would be beneficial to investigate the role of variables such as sex and ethnicity, to supplement these scoring tools, to further understand the impact of these factors on the overall clinical outcomes in CDH. In our study, IS and VIS ≥ 7.5 at 6 HOL was found to have high specificity but low sensitivity in predicting ECMO. These results suggest that IS and VIS < 7.5 at 6 HOL indicate low risk for needing ECMO, but future research should attempt to identify other prognostic variables to discriminate those who will versus will not need ECMO among CDH patients with IS and VIS ≥ 7.5 at 6 HOL. Finally, with the advent of fetal interventions, the impact of FETO on IS/VIS would be an indirect assessment of the impact of this fetal intervention on ventricular function in CDH, which is an area of ongoing research.

## Conclusion

The IS/VIS are objective markers of ventricular function and, with this study, we have correlated these scores at different time points with short term outcomes, and echocardiographic markers of PH/myocardial dysfunction in CDH. This study encourages the use of these scoring modalities in the CDH population and has opened avenues for further research.

## Data Availability

The raw data supporting the conclusions of this article will be made available by the authors, without undue reservation.
